# The Cyclopædia of Practical Surgery

**Published:** 1838-10-01

**Authors:** 


### THE CYCLOPÆDIA OF PRACTICAL SURGERY, COMPRISING A SERIES OF ORIGINAL DISSERTATIONS ON OPERATIVE MEDICINE. By an Association of Physicians and Surgeons. Edited by *William B. Costello*, M.D., Member of several learned Societies, National and Foreign. Part III. Illustrated with Woodcuts. Price Five Shillings, July 1838.

THE part before us of the Cyclopædia of Practical Surgery is equal, indeed superior, to either of its predecessors. Rich both in matter and in illustrations, it offers a favourable sample of the work.

Its contents are:—

Aneurysm, by James Wardrop, M.D.—Aneurysm, local, by Wm. B. Costello, M. D.—Angeioleucitis, by Alf. M. Velpeau, M.D.—Ankle, by Claudius Tarral, M.D.—Ankyloblepharon, by P. T. Dieffenbach.—Ankyloglossum, by Ernst Dieffenbach.—Ankylosis, by John Blackburn, A. M.—Supplement, by W. J. Little, M.D.

### I. ANEURYSM.

The article on aneurysm by Dr. Wardrop is carefully written. It explains the changes which constitute the disease, the history of the operations undertaken for its removal, the effects which such operations produce, and the principles which should guide us in our treatment of the disease. Among other subjects Dr. Wardrop naturally dwells on the operation ultra tumorem, and, perhaps, may look on it with too parental an eye. Be that as it may, the whole article deserves perusal, and it is only the necessary familiarity which our readers must have with many of its details, which prevents us from offering a sketch of it.

But there are a few observations on the *mode of applying the ligature*, which we will take the opportunity of quoting.

In cutting down upon the artery, says Dr. Wardrop, the precise length of the incision in the integuments, after mature consideration, ought to be marked with ink. The length of the incision ought to be regulated according as the vessel may be more or less deeply situated, recollecting that the facility of the future dissection depends very much on a free incision having been made through the skin. The direction of the incision should not be exactly parallel with the line of the artery, but should cross it at an acute angle. This direction of the incision gives the operator the certainty of exposing the artery at one point of his incision; for if it be made parallel to the vessel, a difficulty may arise when the incision does not exactly fall upon the trajet of the vessel. The artery should, of course, be as little denuded as possible.

"This mode of dividing the integuments," continues our author, "offers essential advantages, not only in facilitating the future steps of the operation, but in affording the certainty of denuding the artery at one point, and only at that point where it is desirable the ligature should be applied. For if the operation be executed strictly according to this principle, the cellular sheath of the vessel will be exposed only to such an extent as to facilitate making an opening into it merely sufficient to admit the transit of a small needle for conducting the ligature round the vessel. The safety and success of the operation may indeed be justly said to depend on the ligature being applied without much disturbance to the soft parts contiguous to the artery, and likewise in the facility by which the needle is passed around it; and I am convinced that even the most experienced operators will find decided advantage in performing this operation, by adopting the oblique incision of the integuments which I have now recommended, instead of following the ordinary method of dividing the skin immediately above, and in a line parallel to the trajet of the artery; a practice which I am of opinion it will be better to avoid, even when operating on superficial arteries." 242.

We would observe that this plan is not so universally applicable as Dr. Wardrop would seem to imply. It may be advantageous in operating on superficial arteries, like the radial or the ulnar; in some cases it may be, and it generally is, adopted in operating on deeper vessels, as on the femoral, where the incision, following the course of the Sartorius, cuts the artery at an acute angle; it may be applicable to the carotid above the omohyoideus; but, in the majority of instances, the course of the incision is determined by the course of muscles, the position of tendons, and the constitution of the spaces in which the arteries lie. Take, for instance, the operations on the subclavian—on the iliac—on the tibial—on the brachial vessels. The modern anatomists, and especially Cruveilhier, are particular in insisting on an acquaintance with the direction and the localization of the satellite muscles to the respective arteries. The Sartorius, for instance, is the satellite to the femoral—the biceps to the brachial—the sterno-thyroid to the carotid, &c. These muscles form the guides to the vessels, and these should be always more or less sought for.

"The advantages," continues Dr. Wardrop, "of marking the line of the incision with ink, before commencing an operation, should not be lost sight of, particularly in that of placing a ligature upon an artery. It frequently happens, that, from the external incision not being of sufficient extent, the subsequent dissection is retarded, and it becomes necessary to extend the division of the skin, an inconvenience which can always be avoided by duly considering and marking the length of the incision beforehand." 242.

No doubt this would be advantageous. But unless it is generally done, the surgeon who does it is exposed to observation, and may become the subject of ill-natured jokes.

*Hunter's Claim to the modern Operation for Aneurysm.* Mr. Costello, in the article on Local Aneurysms, and in that part of it in which he treats of the operation for femoral aneurysm, examines this vexata quæstio. As our readers indeed should be aware of the facts, and as the history of the operations for aneurysm is really interesting and instructive, we shall quote it from the researches of the editor of the Cyclopædia.

Lisfranc shows that the first known method for the treatment of aneurysm has been entirely overlooked by every writer on the history of this affection. This method belongs to Rufus, and is thus described by Ætius: *Si vas unde emanat sanguis profundum fuerit . . . , ubi situm ejus et magnitudinem diligenter perspexeris, noverisque numquid vena sit an arteria,* VAS IMMISSA VOLSELLA EXTENDEMUS, ET MODERATE CIRCUMFLECTEMUS. *Ac ubi ne sic quidem cessaverit vinculo constringemus; nonnunquam et post vinculi nexum oblique vas incidere cogimur.* As Mr. Costello justly remarks, this quotation proves, that not only was the idea of employing the simple ligature then known, but even that the torsion of the vessel, which has been of late years described as an invention of modern surgery, was then recommended.

Ætius himself, continues Mr. C., describes another method, which he put in practice for an aneurysm at the bend of the arm. He discovered the artery at four fingers' breadth below the axilla, and applying two ligatures, he then cut the vessel across between them; he next emptied the aneurysmal sac completely, and tied the artery at this point *sicut priorem*, an expression which, according to the interpretation of M. Dézeimiris, seems to say that he also applied two ligatures upon the artery below the tumor.

Paul of Egina's method of treating spontaneous aneurysm, *si ex arteria dilatata tumor obvenerit,* was to expose the vessel by a longitudinal incision, and by means of a needle, pass a double ligature under it, previously plunging a bistouri into the tumor. Though the text is somewhat obscure, it may very naturally be supposed that the ligatures were placed above and below the tumor, and that the sac was opened between them. Here then, observes Lisfranc, is a proof that the method chiefly followed during the 18th century, may be traced back to Paul of Egina. And very probably what Paul wrote others had done. But to proceed.

Avicenna mentions the simple ligature for the wounds of arteries, judiciously recommending that it should be applied between the wound and the heart; and adds, that if the blood should issue from the inferior portion of the wounded vessel, a second ligature should be applied. The only difference between Avicenna's and the modern practice is this:—that, generalising on the facts that have been accumulated, and seeing that hæmorrhage very frequently *does* occur sooner or later from the inferior orifice, we, in all cases where it is possible, tie the artery above and below the wound in the first instance. Mr. Costello goes on to state :—

"Paré, speaking of an aneurism near the shoulder-joint, which had been imprudently opened by a barber, gives the following advice: "Partant je conseille au jeune chirurgien, qu'il se garde d'ouvrir les aneurismes, si elles ne sont fort petites, et en parties non dangereuses, coupant le cuir au dessus, le séparant de l'arteré; puis on passera une esguille à séton, enfilée d'un fort fil, par sous l'artère aux deux costés de la plaie, laissant tomber le filet de soy-même, et ce faisant, nature engendre chaïr qui sera cause de boucher l'artère.'

Guillemeau, a pupil of Paré, gives an account, in the following words, of the operation he performed on the son of M. Belleville: 'Auquel après une saignée faite au ply du bras, lui était survenu un petit aneurisme, qui par succession de temps était accru de la grosseur du poing, auquel enfin le sang contenu en iceluy se groumela; ce qui fut cause d'engendrer quelque commencement de pourriture en ladite tumeur, comme il s'aperçut par le cuir qui avait changé sa naïfve couleur en noirceur et lividité, *estant même altéré et ouvert;* pour à quoi obvier et au grand flux de sang principalement qui s'en pourrait suivre, avec déperdition d'esprits, si l'ouverture se fesait plus ample, je proposai aux médecins et aux chirurgiens le seul remède pour obvier à ce mal, qui était de lier l'artère plus haut que l'aneurisme qui était au ply du bras, à laquelle opinion enfin chacun s'accorda. Premièrement je remarquai sur le cuir l'artère en la supérieure et intérieure partie de l'avant-bras, ainsi qu'elle descend de l'aisselle au ply du bras, trois doigts au dessus d'icelui; et en cette même partie suivant ce que j'avais remarqué, je fis une légère incision en long au cuir, qui était comme séparé à l'endroit de l'artère où elle se rencontre au toucher, et l'ayant ainsi découverte, passai par dessous avec une grosse esguille courbe une petite fisselle desliée, puis avec icelle fisselle je liai ladite artère à double nœud. Cela fait, tout le sang groumelé et autre caillé contenu en la tumeur fut ôté, puis les parois de la tumeur furent lavées avec eau-de-vie à laquelle j'avais fait dissoudre un peu d'Egyptiac pour corriger la pourriture, ja commencée en cette partie; un mois après le malade fut parfaitement guéri sans être aucunement estropié; de quoi j'ai été infiniment esmerveillé.'

The operation which Anel performed a century later, differed from Guillemeau's only in regard to the sac being left untouched; but in both, the ligature was placed on the vessel just above the sac, and it appears that Guillemeau did not by any means consider the emptying of the sac as an essential step of his operation, since he adds to his description of it, 'Si en quelque autre partie extérieure il se présente au chirurgien pareil aneurisme, il peut sûrement découvrir le corps de l'artère vers sa racine et partie supérieure et la lier de même façon *sans autre cérémonie.*'

Now, on neither of these occasions was the principle involved in the Hunterian operation as much as hinted at. The surgeons of the 17th and 18th centuries did not possess that knowledge of the diseases of arteries, which might have led to the adoption of so important an improvement as that with which the name of Hunter is irrevocably identified. Nay, the case operated upon by Dessault in 1785, cannot even be quoted as an illustration of the principle, for Dessault operated on the *popliteal* artery, just above the tumour, as did Guillemeau and Anel on the brachial artery. Hunter tied the *femoral* artery for popliteal aneurism. In so doing, his purpose was obvious: he was aware that the disease in the coats of the artery, upon which the formation of the aneurismal tumour depended, often extended for some distance along the vessel, and he therefore placed his ligature at a distance above the tumour, in order that it might bear upon a sound portion of the arterial trunk, well knowing that the contents of the aneurismal sac might be absorbed. The question therefore is not one of dates, but of facts, and the difference between the two modes of operating, which on one side have been gratuitously assumed to be identical, may be thus summed up: Anel's operation, performed at the beginning of the 18th century, preceded Hunter's by seventy years, led to no result, and was all but forgotten; Hunter's led the way to some of the most brilliant achievements of surgery, and changed the whole practice of our art in regard to this particular class of diseases." 273.

Nothing can be more just than the preceding observations—nothing more illustrative of the value of sound principles. The absurdity of the attempt to rob John Hunter of the merit of revolutionizing the treatment of aneurysm is as palpable to unprejudiced minds as its failure. Guillemeau and Anel tied the vessel been the tumor and the heart, it is true. But they did so at random, without correct reasoning, without sound principle, with indifferent success at the time, and with no effect upon science. To disinter their buried lucky or unlucky accidents, is an envious attempt to rob a great man of justly earned fame, and to nibble at his honest reputation. There is no great modern discovery, which, by a similar process, may not be made to appear an old tale. By a parity of reasoning whatever savage has arrived at the pitch of boiling a kettle, may be shewn to have been an inventor of the steam-engine.

The execution of the whole of Mr. Costello's article is good, and the wood-cuts both numerous and excellent.

### II. Angeioleucitis.*

M. Velpeau, the author of this article, commences by observing that while inflammation of the veins has attracted great attention, that of the lymphatic tissue has been, comparatively neglected. He has long been satisfied of their pathological importance.

> "My attention," he says, "has been devoted to this subject since 1818; and the various papers which I published at subsequent periods, on the alterations of the fluids, are almost as strictly related to it as to the pathology of the veins; the only difference being this—that I had not, as I mean to do now, considered it separately, or in such a manner as to exclude collateral matter. The subject, however, even now, is too novel to admit of its being treated completely, and in all its details. I shall therefore confine myself to the points on which I think I shall be able to throw some light; and on that more especially, which relates to the inflammation of the lymphatic vessels, known under the names of *Lymphangitis, Lymphytis*, &c., and which I prefer designating by the term at the head of this article." 282.

M. Velpeau remarks that the lymphatic tissue is capable of becoming inflamed in various ways and degrees like the other tissues of the body. Inflammation of the lymphatics may arise from injuries of them or of their glands, but usually their diseases result from fluids, either produced or vitiated by inflammation, being introduced therein by absorption or imbibition, and carried from the periphery of the body to the centre.

As the absorbent canals abound in the midst of the organic layers of the body, it is obvious that they must surround and pass through every seat of disease, and that wherever disease exists, they may become charged with heterogeneous materials calculated to inflame them.

The inflammation of the lymphatic vessels presents two distinct varieties. In the one, the seat of the morbid action, or the altered fluids that give rise to it, are protected from the contact of the air; in the other, on the contrary, they are exposed to its action, either primitively or consecutively.

When, continues our author, there is no wound, ulcer, incrustation, or excoriation on the integuments, the altered molecules, modified by inflammation, or by any other morbid action, but rarely acquire properties as deleterious as those which in general characterize them, when deposited in

---

* From ἀγγειον, a vessel, and λευκὸς, white; inflammation des tissues blancs, Fr.; inflammation of the lymphatics.

deep wounds or solutions of continuity; and hence the possibility of their penetrating to some extent into the veins and the lymphatics, without exciting inflammation, or producing any manifest disturbance.

In opposite circumstances, he goes on to state, the contrary obtains; and to be convinced of the fact, we have only to bear in mind, how rapid and numerous are the changes which take place on the surfaces of wounds in all the products of inflammation, in all the substances either taken into or ejected from the body; and that by reacting on each other, under the influence of the air, their elements are transformed, sometimes into new products, comparable in certain cases to real poisons. In becoming fluid, these substances are more in contact with the pores or extremities of divided vessels, and are thus rendered in general, more acrid and penetrating. It seems but natural therefore, that they should obtain an easier access to the torrent of the circulation, and that they should irritate the vessels more highly than in the preceding hypothesis.

"Angeioleucitis may be developed in three different ways: 1. *By continuity of tissue,* or from the external to the internal surface of the canal. In this case the lymphatic vessels that pass through inflamed organs at length become themselves inflamed at the point corresponding to those organs before they exhibit any trace of disease elsewhere. 2. *By obstruction,* or disturbance of their circulation. In this case they are so compressed in the midst of the diseased tissues, that they become inflamed below this point, in consequence of the distention resulting from the stoppage of their circulation; and 3. *By absorption,* or from within outwards. That is, when either, through their lateral pores, or their roots, they take up, in the affected parts, irritating principles in sufficient quantity to determine an inflammation, in the same manner as it occurs in the veins, or when the mischief has its source in a collection seated in the integuments.

In this last case, the inflammation of the lymphatics may also exist in three ways. 1. *By continuity,* or as it takes place in membranous organs; in which case, the inflammation, proceeding from the wound, attacks the lymphatic vessels and seems to be rapidly carried towards their origin or termination, without their becoming of necessity on that account previously charged with the morbific products. 2. *By internal irritation* or by *infection:* that is to say, that those of their mouths or roots, that are enveloped in the diseased locality, becoming charged with heterogeneous molecules, are rendered incapable of bearing the contact of such substances with impunity, and become inflamed secondarily, from the internal to the external surface in one or more points of this course. This is unquestionably the mode in which angeioleucitis is produced in the majority of cases; but we must admit also, that it may proceed from the external to the internal surface, or 3. By *contiguity of tissue,* as in the case where the skin is perfectly entire and healthy." 284.

With regard to the first species of "angeioleucitis," it seems almost sufficient to remark, that inflammation in general, wherever cellular tissue exists, is sufficient to give rise to it.

All kinds of solutions of continuity which either immediately or at a distance communicate with the atmosphere, morbid growths, &c., all conditions of the skin and mucous membrane that produce a morbid change in the fluids which the lymphatics imbibe, frequently determine the second kind of angeioleucitis.

The causes of the affection act with greater energy, that is, it is more common, at puberty and in old age, than at other periods of life. In some cases, says M. Velpeau, there may be absorption to a considerable extent, without producing any manifest bad consequences, whilst, in many others, a few molecules will be sufficient to determine the most intense and extensive inflammation. This peculiarity helps to explain why angeioleucitis is so uncommon, when compared with the frequency of the causes capable of producing it, in the inflammations or other affections not exposed to the action of the air, whilst the slightest puncture or abrasion, the most simple ulceration of the integuments, may quickly produce it. It will also be easily understood how certain relative positions of the orifices of the pores of the lymphatics, as regards deleterious principles, should favour in a particular manner their absorption; vivid moral impressions, a sudden disorder of the functions, in a word, any circumstance capable of subverting for a moment the vital or organic harmony, in parts beyond that which had been primitively attacked, are equally calculated to develop its defects.

M. Velpeau's account of inflammation of the superficial lymphatics is accurate, though diffuse. It is not necessary to lay it before the English reader, who is familiar enough with the characters of the complaint. His account, however, of inflammation of the deep-seated lymphatics will, probably, be new to most practitioners in this country.

"When the attack occurs first in the deep-seated lymphatic vessels, as sometimes happens in the case of wounds or ulcers that pass under the fasciæ, as also of contusion, inflammation, abscess, or ulcer, the symptoms assume a somewhat different character. It is the pain that first attracts notice. It is fixed in a particular spot, where it may remain for a considerable time; it is deep-seated, sharp, or lancinating. If the mischief extends, the pain is soon felt in other regions, simultaneously or successively; and though it cannot be said to be positively absent in the interval separating the points in which it is most acutely felt, still it does not excite sufficient uneasiness to be much complained of. The pain is not radiated in lines from a given point, nor is it diffused, neither does it remit; it is a fixed pain, though disseminated over particular spots, varying in the degrees of its intensity at different points. The tumefaction comes on afterward, or it may occur almost at the same time, and in the same places as the pain. It proceeds, and is developed from the centre to the circumference. It is observed under the form of masses of more or less extent, of thick and long cores; but there is no appearance of patches, as in the case described already. Its origin is without difficulty recognized to be from beneath the fascia, and the tissues are less dense in proportion as they are nearer to the skin, which retains for a long time suppleness and a certain degree of mobility. Should the swelling become general, the first characters will nevertheless persist to some extent to the end; in the midst of the general tumefaction we can discover, here and there, points more swollen and perceptibly more dense than others. The redness only becomes manifest subsequently to the two preceding phenomena. It is less superficial than in the former variety, and can be only seen as it were by transparency in the shape of irregular patches, and not as streaks or bands. It would seem to be more intense and more extensive according as the eye pursues it to a greater depth. The skin moreover being stretched, and as it were thinned, is shining, and more of a whitish or pale rose colour, than truly red, in the intervals of the inflamed points. Generally speaking, it is the deep lymphatic ganglions that first swell and become painful. The inflammation proceeds so rapidly, and to such an extent, that the deep-seated variety of angeioleucitis has, in many cases more the appearance of inflammatory œdema, than that of a diffused phlegmon or of a phlegmonous erysipelas." 286.

We must confess that we are not quite satisfied of the absolute correctness of the preceding account. It seems to us that M. Velpeau has partly described diffuse inflammation of the deep-seated cellular tissue, partly that of the deeper lymphatics. But to proceed. He remarks that the two varieties run into one another, and that they do not long remain perfectly distinct. The superficial and deep-seated glands almost invariably become affected in both cases.

M. Velpeau presents an explanation of the appearances and progress of the inflammation of the absorbents. He then gives an account of the general symptoms. But it is not necessary to follow him in his remarks.

Inflammation of the lymphatics may terminate in resolution, suppuration, induration, or death. Of the termination by resolution it is not necessary to speak. Suppuration, says M. Velpeau, must be expected to supervene whenever the red patches are numerous, and have a tendency to run into each other, and when beneath them, painful cores of a certain thickness can be felt. The suppuration, however, takes place slowly, and is found in two different states, either in that of infiltration, or as a collection of variable extent. The pus remains infiltrated under the red streaks, along the vessels, and between the layers of muscles. The abscesses occur more particularly under the patches, and in such a manner that the principal inflamed nuclei form the centres of so many purulent collections. Sometimes, also, the abscess is large, and the matter is diffused as in the case of a phlegmonous erysipelas. But whether these abscesses be superficial or deep-seated, the fluctuation is not felt in them till an advanced period, and when opened, the quantity of pus which escapes is much more considerable than could have been expected from the apparent extent of the tumefaction. There is generally more than one, and the first collection is merely the precursor of several others, which occur in succession, at intervals of a few days, and at different points. M. Velpeau has seen as many as twenty-five in the same individual. They are surrounded, he continues, by a pasty, or hardened edge; but it does not remain so long as in the case of a common phlegmonous abscess. In the cavity, neither bands nor partitions can be detected, and although the pus contained is usually very fluent and thin, the secretion soon ceases, and the parts cicatrize rapidly.

The termination by induration, without suppuration is rather uncommon, and occurs almost exclusively in chronic angeioleucitis.

The complaint may terminate in death, either when the inflammation is at its height, or from prolonged suppuration. In the latter case the fatal issue may result from the exhaustion produced by profuse purulent secretion, from the occurrence of fresh attacks of the disease in the viscera, or from effusion into the serous cavities, and vitiation of the blood, by its mixture with the pus, or absorbed substances.

"On the whole," says M. Velpeau, "the progress and duration of angeioleucitis are extremely variable; in some instances, it is developed so rapidly that on the eighth day there can be no longer a doubt as to the formation of pus; in others, the progress is so slow, that even on the twentieth day, there is no saying with certainty how it will terminate. In some individuals its phases are perfectly regular from beginning to end, whilst in others, all the groups of symptoms are graduated and distributed as if they belonged to several distinct and successive inflammations. When resolution takes place, it may be looked for from the fourth to the tenth day. The suppuration may be established as early as the eighth day, but most commonly it does not become manifest until the fifteenth or twentieth. It is also from the eighth to the twentieth day, that death is observed to take place. Beyond this period it seldom occurs till after the thirtieth or fortieth day, when induration may take place, and when internal collections, the contamination of the blood, and diarrhœa, are more especially to be feared." 288.

After some further observations on the circumstances that render angeioleucitis more formidable, or less so, M. Velpeau sums up by admitting that nothing can be more difficult than to generalize on the prognosis of the affection.

M. Velpeau proceeds to point out the distinctive marks of angeioleucitis and of other affections with which it may be and has been confounded—phlebitis, erysipelas, neuritis, phlegmonous erysipelas, and erythema nodosum.

*Phlebitis.* The red bands, observes our author, which indicate it are larger, fewer, less frequently crossed, more deeply seated, and correspond to so many hard, round, moveable cords, which are painful, and sometimes as thick as the finger. The red patches, when any exist, rest on nuclei that are more clearly defined, less deep, and less adherent; and they seldom become united in such a manner as to constitute a tolerably regular erysipelas. The suppuration occurs more rapidly, and the abscesses thus formed are either diffused, as in phlegmonous erysipelas, or less full of matter than might be supposed before they were opened. The pus evacuated from them in the latter case is often reddish, and seems as if it were mixed with decomposed blood. They are sometimes very numerous, but smaller; and they are almost always developed along the tracks of the large venous trunks, but not dispersed indifferently throughout the different regions of a limb. The tumefaction is, in general, less, and does not extend to the entire thickness, or to the whole circumference of the part, unless the phlebitis be deep-seated, and in that case, the redness of the skin, and the subcutaneous abscesses have no resemblance whatever to those produced by angeioleucitis. The skin is neither tense nor shining; it seems rather thickened, or simply inflamed. We cannot agree with M. Velpeau in the assertion that, in phlebitis, the skin is neither tense nor shining. The tension and gloss of the integument are merely accidental circumstances. They depend on deep-seated effusion which puts the skin upon the stretch, without any inflammation of the latter, or of the subcutaneous tissues. In general, adds M. Velpeau, the lymphatic glands are neither swollen nor painful in phlebitis.

The general symptoms of phlebitis are more of a typhoid, those of inflammation of the lymphatics, more of an active inflammatory type. But there are exceptions to this rule. Phlebitis is more prone to infect the constitution and give rise to the symptoms and consequenes of the absorption of pus, than inflammation of the lymphatics is. We think M. Velpeau is right when he remarks, that—phlebitis, in a word, is formidable, more particularly from the kind of poisoning it causes, and by the facility it finds of extending itself towards the great vascular trunks; while angeioleucitis is infinitely more so, on account of the local disorders of a decidedly inflammatory nature that result from it. As a local inflammation, the former is very easy, the latter very difficult to check. As a general affection, the case will be exactly the reverse of this.

As phlebitis may supervene on angeioleucitis, or the latter on the former, and as the veins and absorbents are very analogous in structure and in functions, no absolute line of distinction can, after all, be drawn between their inflammatory affections.

*Neuritis.* The symptoms of this and of angeioleucitis are so obviously different, that we think it quite unnecessary to enter on the diagnosis of the two.

*Erysipelas.* M. Velpeau observes that angeioleucitis is more frequently mistaken for erysipelas than for any other affection. This is chiefly because many surgeons confound, under the name of erysipelas, many different diseases.

Common erysipelas, he goes on to say, is neither preceded nor accompanied by red streaks, or tumefaction of the lymphatic ganglions. If the contrary should take place, it only so happens in consequence of the erysipelas being complicated with adenitis, or angeioleucitis. The inflammation in this case seems to proceed from the epiderm towards the deep-seated layers of the skin, and is not manifested in scattered patches. The subjacent swelling is equal, and simply subcutaneous, being neither deep-seated nor distributed in masses or cores. It extends by continuity of tissue, instead of bounding, as it were, from point to point, over different regions. The skin, and not the layer beneath it, is its primitive and specific seat. Its progress is much more rapid: the formidable symptoms, such as delirium, and dryness of the tongue, often appearing as early as the fourth or fifth day. It rarely terminates in suppuration or induration, and resolution takes place from the third to the eighth day. When death occurs, it can scarcely ever be attributed to the local mischief.

In erysipelas, he continues, the surface of the epiderm seems as if thickened, and presents small vesicles, and sometimes even phlyctenæ of a certain size. It has a yellowish rosy hue, terminating abruptly at the limits of the disease, by an elevated and festooned edge; half a line beyond which, the slightest alteration of colour in either the skin or epiderm cannot be discerned, and it would seem as if the inflammation was shed over the derm like a layer of milk or oil. We see nothing of this kind in angeioleucitis,—the redness of which is bright or pale, or inclined to violet; it has no defined limit, but disappears insensibly, in such a manner, that it would be impossible to fix the line that separates the disease from the unaffected tissues. The skin may be thickened and elevated, but in general it is not covered, either with vesicles or phlyctenes at the commencement; nor does it present a surface in relief, as if it had been overlaid with another layer.

Phlegmonous erysipelas presents differences less strongly marked.

"At the outset, most commonly, the general symptoms are absent; the inflammation spreads gradually, leaving none of the parts unattacked through which it passes. It does not appear under the form of scattered patches, or indurated cores; neither is there any appearance of red or livid bands. The ganglions are not engorged, nor does it seem to be developed more along the trunks of the lymphatics, than elsewhere, although, like angeioleucitis, it appears to spread rather from the deep-seated tissues towards the surface, than from the epiderm towards the fasciæ. Suppuration takes place very rapidly, the collection being large and widely diffused, and the pus greyish and thin. The cellular tissue is extensively destroyed, and the integuments are so rapidly thinned as to be in danger of perishing also. If the inflammation has not attacked from the beginning a considerable extent of surface, the febrile reaction will be, in general, trifling before the pus is formed.

The general symptoms are not of a nature to excite any serious alarm, till after the second period. We then have symptoms of infection, but which tend rather to adynamic than to the ataxic character. The skin becomes dull, soiled, and earthy; the tongue blackens, and the delirium is of a calmer kind." 290.

But the two diseases are frequently complicated with one another. This occurred to a remarkable extent in M. Velpeau's own wards, in the months of March, April, and May, 1837. Almost all the patients that had been affected with angeioleucitis were soon after attacked with erysipelas, strictly so called, or with phlegmonous erysipelas; and, in like manner, those who were first attacked with erysipelas, soon presented it complicated with angeioleucitis.

Of *Phlegmon* and *Erythema Nodosum* we do not think it necessary to speak.

It seems to us, from a perusal of M. Velpeau's observations that they are open to two criticisms:—first, he does not appear to be aware of the extent to which inflammation of the absorbents is understood in this country; secondly, we cannot help suspecting that he himself confounds to a certain degree diffuse inflammation of the *deep cellular membrane* with inflammation of the absorbent vessels. But however this may be, the present paper merits the attentive examination of surgeons.

*Pathological Anatomy.* M. Velpeau arranges the alterations that result from inflammation of the lymphatic vessels, under three heads:—the first is of the vessels themselves; the second takes place in the interposed tissues; the third must be sought for in the viscera, in the blood, and in the remote regions.

When the absorbent vessels themselves can be successfully made out, their internal surface appears of a milky colour. On the outside, they are surrounded with cellular tissue, which is easily broken down, and more or less infiltrated with a turbid, half-concrete lymph; their parietes are evidently thickened: yet even still they may be permeable.

"The points most diseased are those which correspond to their crossings, and those opposite to their valves; it is at these points that their envelope is often found infiltrated with true pus, that they are closen, that lardaceous cores are observed, the same as in the centre of an abortive phlegmon, and that we must look for the origin of some of those abscesses, that have been observed during the progress of the disease. The skin, which is sometimes covered with large phlyctenes, presents towards the end scattered eschars, or mortified patches. These patches are greyish, or of a yellow white; they are softened, puffed, and in a state of purulent decomposition, or rather of gangrene,—their aspect bearing some analogy to the soft tenacious centre of the furuncle or anthrax. Beneath the integuments, the cellular tissue is found to possess a healthy character in some places; in others, it is more or less indurated, and, as it were, lardaceous, infiltrated with pus or turbid serum, broken up by ulceration wherever purulent collections have taken place, and more or less thickened throughout. The fasciæ, muscles, and nervous cords are but little altered, the disorders being confined chiefly to the interstitial cellular tissue. Between the muscles, around the vessels, in a word, wherever this tissue exists, it is infiltrated, hardened, thickened, or destroyed, from space to space, in the same manner as we observe under the skin. When there is suppuration, the matter is found in circumscribed collections, rather than in sinuses, or canals; and, unless in a very rare case, no mortified tissue can be discovered beneath the fascia. The arteries and veins are sometimes thickened or increased in size: but in dissecting them, this appearance is found to depend on the thickening of their external layers, and on their vicinity to the larger lymphatic vessels.

If the disease has existed for any time, the blood is in general very fluent, abounding in serum of a reddish rather than a dark colour. The few coagula which are found in the nervous system are diffluent, and frequently mixed with yellow grains. When polypiform concretions are found in the arterial system they are also more pliable than those met within persons who die of diseases purely inflammatory; they are also less homogeneous, and they frequently present yellow, black, white, and red fasciculi. Pus, however, has not been found either in the ventricles or the great vascular trunks." 291.

Abscesses are rarely discovered in the great parenchymatous organs. But they or their serous membranes may be affected with the secondary inflammations. On the whole, sums up M. Velpeau, the internal lesions found after death are often very disproportionate to the symptoms observed during life. The same remark is not wholly inapplicable to the local lesions; for we often find that the traces of inflammation are slight where the angeioleucitis had appeared to be severe.

*Treatment.* M. Velpeau starts with a proposition which is almost self-evident, that, in order to appreciate the value of the various modes of treatment recommended for angeioleucitis, we must first have learnt to distinguish it from other affections, and its various shades from each other. We shall consider his recommendations seriatim.

1. "General bleeding, which is proper in the commencement when the patient is young and robust, and there is fever present, is useless when the reaction is slight; and generally hurtful, when the period of suppuration has arrived, and at all times in the majority of aged persons, or of those whose constitutions are worn out. Leeches applied in considerable numbers over the sites of the inflammation, are either useful or hurtful under similar circumstances. When applied towards the root of a limb, in the vicinity of the engorged ganglions, they will be often found sufficient, for this simple reason, that rest, regimen, and simple external applications will cure angeioleucitis in a vast number of cases, and when attacked in the first stage." 292.

The latter observation is perfectly true, indeed all the preceding directions are so. It may, we think, be very fairly stated, that one half of the remedial means we possess owe their supposed efficacy to the curative powers of Nature; that is, they are applied in cases which tend to get well of themselves. Certainly this explanation will fit a good many of the remedies for inflammation of the absorbents.

2. "Emollient poultices applied over the solutions of continuity, and compresses of the same kind over the erysipelatous streaks, which are the only proper topical applications, while the patient is kept to the antiphlogistic plan, are of themselves but of little value when the disease is deep and extensive. Purgatives, in the beginning, aggravate the mischief; they are useful only in the subsequent stages, and then merely as accessory means." 292.

Although we have seen pressure of service, we should be sorry to recommend it as indiscriminately as M. Velpeau seems to do. We are satisfied that there are many cases in which it would be mischievous, nay, we have ourselves seen it so.

In M. Velpeau's remarks on purgatives we discern both the prejudice of the French school, and an apparent inconsistency. Why purgatives should be useful in the latter stages, when the inflammation must have declined or terminated in suppuration, and useless in the early stage, when the inflammation is coming on or at its height, we profess ourselves unable to discover. As a matter of practice, and it is borne out by reason, we are sure that purgatives are very valuable in the onset of this, as of other inflammatory affections; indeed, judiciously administered, they are valuable throughout.

3. " I have tried blisters; when employed in the usual way, they are altogether incapable of extinguishing or stopping the inflammation. Their action, when applied over the prominent points of the indurated masses, is limited to the hastening, or determining the suppuration or the resolution, which till then seemed uncertain. When, on the other hand, I have employed them to an extent that would really seem monstrous, the effects produced have been almost miraculous. I am not afraid, for example, to envelop the whole limb, from the shoulder to the fingers, or from the groin to the knee, and from the knee to the toes. The general and local symptoms are, by this treatment, arrested so rapidly, that in a week or two, a resolution which could scarcely have been expected, is effected. This plan of treatment may justly be considered as heroic." 292.

We would observe, in reference to this, that inflammation of the absorbents being a complaint which in a very large proportion of cases tends to run a favourable course spontaneously, we should be chary of severe remedies. When the attack is really formidable, M. Velpeau's observation on blisters should be borne in mind. But this must not be forgotten:—the value of mercury, antimony, and so forth, not being appreciated on the Continent, such remedies as blisters obtain undue credit. They carry off in the shape of serum, fluids which we discharge by the bowels, and whilst our means of revulsion tell upon the liver, the skin, the alimentary canal with its appendages, our foreign confreres confine themselves from timidity to the more gross, more severe, and often less efficient modes of depletion or cutaneous counter-irritation.

4. M. Velpeau justly observes that usually incisions are only necessary when suppuration has occurred.

Passing over some other observations, we shall conclude by quoting the plan of management which M. Velpeau adopts and recommends.

" I subjoin the plan of treatment which has appeared to me to be the most successful. If any wound exist, whether it be recent or of long-standing, it is covered with a thick poultice: if there be any arterial reaction, recourse is had to general bleeding; the quantity abstracted to be somewhat considerable; to be followed up by the use of a tepid bath for an hour; twenty or thirty leeches are applied round the wound, when it appears red and swollen. After these means, compression with a roller will be of use; its action will be promoted by wetting it several times during the day with some resolvent fluid. Cold water perhaps would answer very well in this way. If compression is not followed by the desired effect, mercurial inunction is indicated. The plan I pursue is to have two drachms rubbed in three times in twenty-four hours, over the entire extent of, and a little beyond, the painful region. When the skin becomes surcharged with the ointment, it is easy to clear it again by means of a little oil, and then a second bath is taken. As soon as any fluctuation can be detected, no matter how obscure at any point, the bistouri must be made use of at once, all these abscesses requiring to be largely and promptly opened. At this stage, recourse may again be had to poultices over the suppurating points, and to compression, if the form of the part will allow it. When resolution does not take place, and the suppuration is tardy, it will be right to employ blisters. They may be applied in succession, or simultaneously over the points where the inflammation was most intense, and which are still the most engorged. The larger the blisters, the more certain their good effects, acting either as the best maturatives, or the best resolvents that I know of. In many such cases, I can speak decidedly as to their good effects; having succeeded, in a little time, by means of them, either in dispersing or producing suppuration in masses which seemed to be unalterably indurated. When there is delirium, or great agitation, I also employ them from the beginning; arranging them in such a manner as to cover over the whole of the diseased, together with some of the sound parts. They are removed in twenty-four hours with the epiderm, and the exposed parts are powdered with camphor, in order to protect the urinary passages. The dressing is completed with simple ointment spread on bibulous paper. Mercurial inunction and compression may be conjoined with these means after a few days.

A purgative every third or fourth day, for a week or two, towards the decline of the disease, will be found useful. But medicines of this class should be carefully avoided if diarrhœa or other signs of visceral irritation be present. At a still later period, when the wounds cease to suppurate, it is sometimes imperative to have recourse to friction with the ioduretted ointment, on account of the doughyness and induration which often persist in several points. Frictions, with small quantities of mercurial ointment, deserve the preference when the induration is extensive; and compression would be still better when it is possible to apply it. The use of the tepid bath is of great value. It need scarcely be added, that from beginning to end, the parts should be kept at rest, and that the regimen and diluents should be regulated according to the state of the bowels, and the degree of general reaction. The wounds, ulcers, and eschars resulting from angeioleucitis require the same treatment as under any other circumstances." 293.

We cannot say that we have any great amount of faith in mercurial inunction. When the inflammation is of a sthenic character, we have found evaporating lotions or plain bread poultices as good local applications as any. When the inflammation is of an asthenic character, the patient old or weak, or suppuration impending, fomentations or warm poultices are advisable. In some cases there is a great tendency to the effusion of serum or semi-solid lymph into the cellular texture. Those cases have appeared to us the ones, in which moderate compression by means of strips of calico, spread with cerate, has answered best.

This article has extended too far to permit us to carry it to greater length. The remaining papers in the part of the Cyclopædia of Surgery before us are carefully compiled. We would however recommend a little more attention to the *composition.* A bastard sort of English has lately been adopted by our Cyclopædia writers, which, if not repressed, bids fair to debase our noble language in its application to medicine. A quartum quid between French, German, and English, would appear to be in process of formation, repugnant to men of general education, and barbarous to the greatest degree in itself. We trust this will be corrected.

